# Vowel speech recognition from rat electroencephalography using long short-term memory neural network

**DOI:** 10.1371/journal.pone.0270405

**Published:** 2022-06-23

**Authors:** Jinsil Ham, Hyun-Joon Yoo, Jongin Kim, Boreom Lee

**Affiliations:** 1 Department of Biomedical Science and Engineering (BMSE), Gwangju Institute of Science and Technology (GIST), Gwangju, South Korea; 2 Department of Physical Medicine and Rehabilitation, Korea University Anam Hospital, Korea University College of Medicine, Seoul, South Korea; 3 Deepmedi Research Institute of Technology, Deepmedi Inc., Seoul, South Korea; Federal University of Rio Grande: Universidade Federal do Rio Grande, BRAZIL

## Abstract

Over the years, considerable research has been conducted to investigate the mechanisms of speech perception and recognition. Electroencephalography (EEG) is a powerful tool for identifying brain activity; therefore, it has been widely used to determine the neural basis of speech recognition. In particular, for the classification of speech recognition, deep learning-based approaches are in the spotlight because they can automatically learn and extract representative features through end-to-end learning. This study aimed to identify particular components that are potentially related to phoneme representation in the rat brain and to discriminate brain activity for each vowel stimulus on a single-trial basis using a bidirectional long short-term memory (BiLSTM) network and classical machine learning methods. Nineteen male Sprague-Dawley rats subjected to microelectrode implantation surgery to record EEG signals from the bilateral anterior auditory fields were used. Five different vowel speech stimuli were chosen, /a/, /e/, /i/, /o/, and /u/, which have highly different formant frequencies. EEG recorded under randomly given vowel stimuli was minimally preprocessed and normalized by a z-score transformation to be used as input for the classification of speech recognition. The BiLSTM network showed the best performance among the classifiers by achieving an overall accuracy, f1-score, and Cohen’s κ values of 75.18%, 0.75, and 0.68, respectively, using a 10-fold cross-validation approach. These results indicate that LSTM layers can effectively model sequential data, such as EEG; hence, informative features can be derived through BiLSTM trained with end-to-end learning without any additional hand-crafted feature extraction methods.

## Introduction

Speech carries vast amounts of information to the brain, and it is one of the typical features of the brain to recognize and categorize the sounds of behaving animals. Given its importance, attempts to investigate the mechanisms of speech sound recognition have been conducted for over 100 years. One of the first neurolinguistic study of speech recognition was conducted through an observational study in the 1870s by a German neuropsychiatrist who found the crucial role of the superior temporal gyrus in speech perception, deducing that deficits in speech recognition were associated with damage to the left superior temporal gyrus [1]. It is now known that speech recognition relies predominantly on the dorsolateral temporal lobes, including the superior temporal gyrus, which contains the primary auditory cortex (A1) and anterior auditory field (AAF) [2]. Although the manner phonemes are encoded and interpreted in the brain remains controversial, it has been widely accepted that the recognition of sound is categorical. That is, discrimination is better for stimuli belonging to different phonetic categories than for stimuli belonging to the same category, even if the acoustic differences are equivalent [3, 4]. Not only humans, but also animals’ perceptual systems sort continuously varying sound stimuli into a set of discrete categories [5].

With the advances in neurophysiological studies, electroencephalography (EEG) has been widely used in research involving neuroscience and neural engineering [6]. The high temporal resolution and sensitivity to different functional brain states make EEG a powerful tool to investigate real-time brain activity, and there has been increasing interest in illuminating the neural basis for categorical perception. Traditionally, EEG signals are recorded non-invasively from scalp in human study. At the level of sound or speech perception, mismatch negativity (MMN), a component of auditory evoked potential (AEP), which is elicited by oddball sounds, is widely used to study neural correlates of categorical perception [7, 8]. Naatanen et al. found evidence for language-dependent vowel representations in the human brain [9]. Another study examined the categorical perception of lexical tones and found that across-category contrast elicited a larger MMN than within-category distinction [10]. In animal experiments, more accurate EEG signals were obtained through invasive procedures. For instance, neural correlates of categorical perception and neural representations of various sounds have been studied using extra-cellular recording of action potential. Striatum-projecting neurons of song birds display categorical auditory responses and are highly sensitive to changes in note duration [11]. In addition, Kilgard et al. studied distinct neural representations of consonant and vowel sounds using intraparenchymal recording in the rat brain. Recording the multi- and single-unit responses from the inferior colliculus and A1, they suggested that the spike count encodes vowel sounds, while spike timing encodes consonant sounds [12, 13]. The effects of sound discrimination training in a rat model of autism were also investigated based on previous findings correlate neural responses to sound stimuli with sound perception ability [14]. Moreover, a recent study demonstrated that electrocorticography recorded with multi-channel array correlates with a passive exposure to a specific sound even in the auditory cortex of anesthetized rats [15].

Machine learning approaches have been used to make practical use of EEG in a wide variety of studies. Utilizing machine learning methods enables the investigation of rich information that is inherent and difficult to uncover from EEG signals [6]. Therefore, EEG-based classification can be performed in the following fields through conventional machine learning algorithms (e.g., support vector machine (SVM), k-nearest neighbors (KNN), and naïve Bayes (NB)): motor imagery, emotion recognition, mental illness detection, event-related potential (ERP) detection, and so on [16, 17]. Furthermore, in recent years, owing to the increasing advances in graphic processing units and the availability of large dataset, it has become possible to conduct EEG-based classification using various deep learning networks [6, 18, 19]. Compared with conventional machine learning methods, deep learning networks are able to automatically detect and extract appropriate representations from input data [20, 21]. Hence, even with insufficient prior expert knowledge, promising results can be obtained through deep learning algorithms that do not require an additional handcrafted feature extraction process [22, 23]. For example, in the field of speech, images, and video, the results were significantly improved by applying deep learning algorithms [24–26]. However, it is not clear whether such outperforming results always accompany the EEG-based classification domain when utilizing deep learning approaches instead of traditional machine learning methods [27]. Roy et al. showed that in most of the studies (excluding four out of 102 studies), the deep learning approach led to a higher performance than the traditional machine learning approach, and the highest improvement in accuracy was 35.3% [18, 28].

Furthermore, among the various fields of EEG-based classification studies, ERP classification studies are actively conducted by applying both conventional machine learning and deep learning methods. In an early study, the traditional grand averaging method was utilized to improve the low signal-to-noise ratio (SNR), one of the limitations of EEG signals, and to obtain ERP signals. In these studies, several ERP components were treated as feature sets for classification [29, 30]. In animal studies, the ERP features such as peak amplitude and latency are also used to discriminate ERP signals [31, 32]. However, single-trial EEG-based classification has also received much attention, since it is known that EEG data at the single-trial level possess more functional and rich information than the ERP signals obtained through the traditional grand averaging method [33, 34]. Therefore, in subsequent studies, features extracted by various algorithms such as wavelet-based algorithms [35], Gaussian mixture models [36], and spatial filtering [37] for classification using conventional machine learning methods [38, 39]. However, extracting the optimal hand-crafted features from the single-trial EEG is time-consuming and labor-intensive because additional processing steps must be executed. In this context, deep learning methods can alleviate this problem by allowing end-to-end learning. The most prevalent deep learning architecture is convolutional neural network (CNN), followed by recurrent neural network (RNN). The CNN is a special type of deep learning architecture widely used for single-trial EEG-based classification [6]. The CNN inputs are derived from raw or preprocessed EEG data, primarily in the following form: number of channels × number of time points in a single trial. Moreover, considerable classification results have been demonstrated and it has been known to perform best when using spectrogram images as inputs [40–44]. In contrast to CNN, RNN is a highly preferred architecture, especially when handling sequential data (as in natural language processing applications) because the recurrent connection of RNN learning architecture makes it possible to utilize the previous information of the network recursively as the current input data [45]. Long short-term memory (LSTM) is a kind of RNN architecture proposed by Hochreiter and Schmidhuber to overcome the exploding and vanishing gradient problems of RNN [46]. Bidirectional LSTM (BiLSTM) is a further development of LSTM that combines the forward and backward hidden layers to access both the preceding and succeeding information. Although BiLSTM model is much complex and might need additional computational power, it is expected to solve the sequential modelling and classification task better than LSTM [47].

Previously we tried to classify EEG signals on a single-trial basis for three vowel sounds, /a/, /o/, and /u/, using machine learning techniques for the human brain. After the application of appropriate signal processing algorithms, including multivariate empirical mode decomposition (MEMD), the EEG responses were effectively classified according to each vowel sound using a linear discriminant analysis (LDA) classifier. From the time-frequency representation (TFR) of the EEG signals, it was also determined that the alpha band components were the most related neural responses of vowel sound perception [48]. However, due to the low SNR of human EEG signals, phoneme representation in the brain needs to be further assessed with a more invasive recording technique, allowing the acquisition of more reliable EEG signals. In addition, it is necessary to conduct further studies on the classification performance of each machine learning algorithm in classifying EEG responses to different phonemes.

The primary purpose of this study was to determine specific EEG components that might be related to speech representation in the rat brain to further illuminate brain responses to speech sound recognition. To acquire more accurate EEG signals, epidural EEG signals in response to auditory stimuli were recorded in AAF, which has been known to play an essential role in auditory perception and categorization [2]. In addition, this study tried to discriminate different brain responses for each speech sound on a single-trial basis using LSTM networks and other conventional machine learning techniques. It was hypothesized that the BiLSTM network would be appropriate for classifying EEG responses to vowel stimuli and would outperform other classical classifiers, because the network can perform robustly in modeling long-term dependencies of sequential data such as EEG. To the authors knowledge, LSTM networks have not been applied to the classification of EEG responses to auditory stimuli, and this is the first study to use a deep learning algorithm to analyze epidural EEG signals from AAF. Moreover, using the deep learning algorithm, EEG responses were classified to auditory stimuli using end-to-end learning with minimally preprocessed EEG signals with no additional feature extraction methods.

## Materials and methods

### Animals

The minimum required sample size was calculated to be 11 to 19, referring to previous animal studies that characterized neural responses to different human syllables [12, 13, 49]. Considering both scientific validity and animal ethics, a total of 19 male Sprague-Dawley rats (325–400 g, 11–13 weeks of age at the time of the experiment, Orient Bio Inc., Seongnam, Korea) were enrolled in the study. Only male rats were included in this study to avoid the potential effects of estrogen on EEG [50]. The animals were individually housed in standard plastic cages with free access to food and water and were maintained at a constant temperature (21 ± 1°C) with a 12 h light/dark cycle. All experimental protocols and procedures were approved by the Institutional Animal Care and Use Committee (IACUC) of the Gwangju Institute of Science and Technology (GIST). According to the committee, the study belonged to United States Department of Agriculture Category D; pain or distress was appropriately relieved with anesthetics, analgesics and/or tranquilizer drugs or other methods of relieving pain and distress. Therefore, all the surgical procedures and animal care were carried out in accordance with their guidelines to ensure minimal discomfort to the animals (approval number: GIST-2019-047).

### Surgical procedures

All rats underwent microelectrode implantation surgery to acquire EEG signals in response to the speech sound stimuli. Before the surgery, the rats were anesthetized with isoflurane (5%) mixed with oxygen gas (0.6 L/min flow rate) in an induction chamber. Once the rats lost the righting reflex, they were moved into a stereotactic frame and applied an anesthetic nosecone. Isoflurane gas (maintenance dose of 1.5%) mixed with oxygen was redirected to the nosecone. Next, ear bars were inserted into the ear canals to fix the head. We then shaved the fur from the ears to just between the eyes. A line block with 2% lidocaine was performed on the scalp, and an incision was made to expose the skull. Next, the bilateral temporalis muscles were partly removed and durotomy was performed on each AAF with a dental drill to insert the epidural EEG electrodes. The electrode was a single micro-electrode that was custom-made using a micro-screw, silver wire, and a connector. The coordinates of the AAF were as follows: 4 mm posterior, 7.6 mm lateral, and 4 mm ventral to the bregma [51]. Finally, the implanted electrodes were connected to a multi-pin connector and fixed to the skull using bone cement. After completing all the surgical procedures, the rats were injected with an antibiotic (ceftazol 20 mg/kg, Guju Pharma Co, Korea) and an analgesic agent (ketoprofen 2.5 mg/kg, Uni Biotech, Korea) intramuscularly for three consecutive days. All animals were allowed to recover for a week and closely observed for any signs of pain such as reduced appetite, hunched posture, or piloerection.

### Speech stimulation

Frequency information of speech sounds is known to be essential for categorical perception and recognition of different vowels [52]. In addition, components of AEP vary according to sounds with varying frequencies, and these different brain responses can be used to study sound recognition mechanisms [9]. Therefore, five different vowel speech sounds, /a/, /e/, /i/, /o/, and /u/, which have very distinct formant frequencies for each speech stimulus were chosen [53].

**Fig 1:**
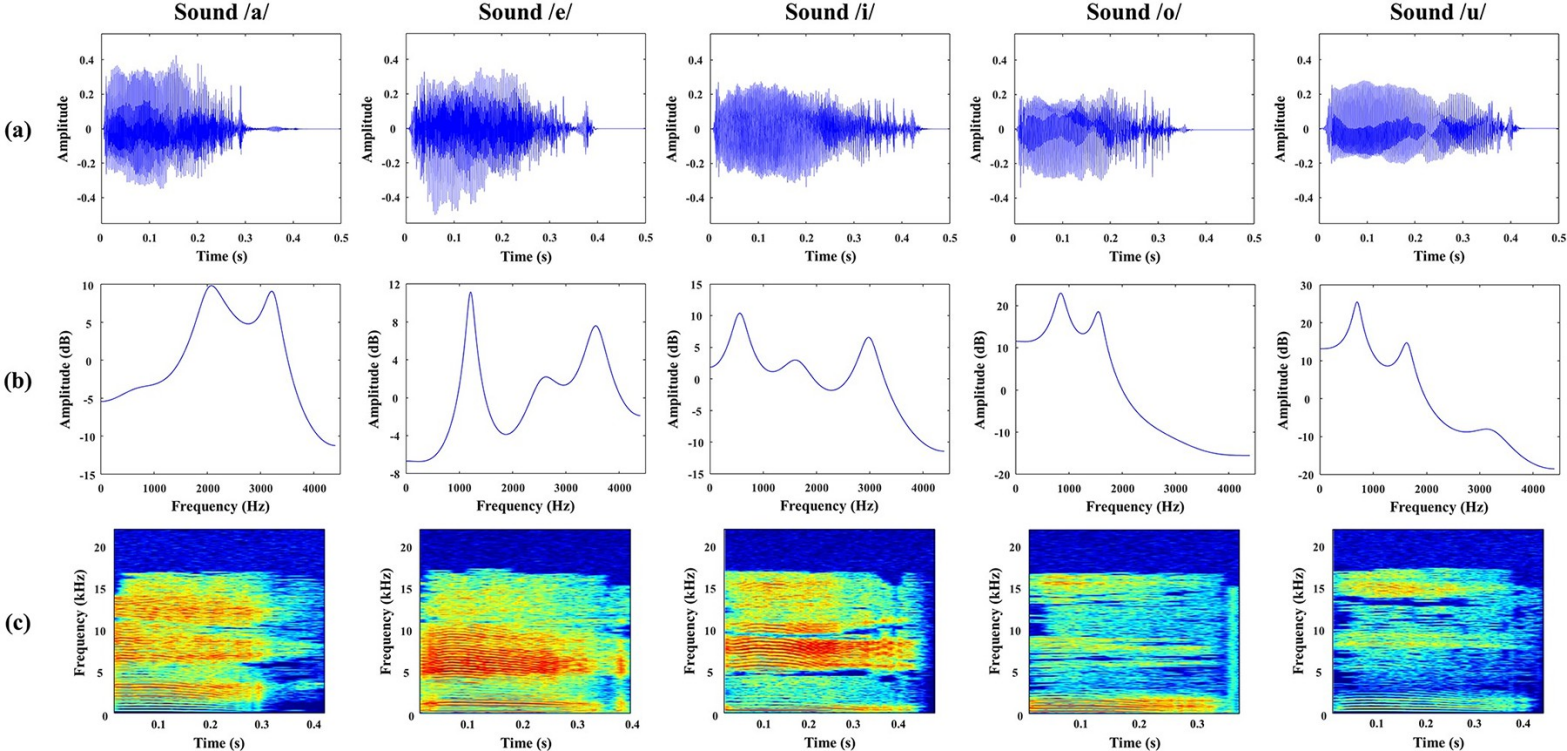
Characteristics of each vowel speech sound. (a) Time course, (b) linear predictive coefficient (LPC) spectra, and (c) the spectrogram of five vowel sounds used in this experiment. The peaks of LPC spectra refer to the formant frequencies of the sound stimuli. Vowel /a/ shows peaks at F1 = 651 Hz, F2 = 2034 Hz, F3 = 3234 Hz; vowel /e/ at F1 = 1211 Hz, F2 = 2559 Hz, F3 = 3570 Hz; vowel /i/ at F1 = 559 Hz, F2 = 1630 Hz, F3 = 2988 Hz; vowel /o/ at F1 = 845 Hz, F2 = 1564 Hz, F3 = 2921 Hz; vowel /u/ at F1 = 699 Hz, F2 = 1636 Hz, F3 = 3299 Hz.

All speech stimuli were generated using a text-to-speech program provided by Google and the sound pitch was increased by one octave using the shiftPitch function in MATLAB 2017b (Mathworks, Inc., MA, USA) to accommodate the rat hearing range and applied root mean square normalization. The stimuli were delivered by a speaker (SRS-X88, SONY Co., Japan), which was located above one side of the cage, approximately 15 cm from the rat’s head and the maximal intensity of the sound was calibrated to 60 dB SPL. The vowel speech sound was analyzed according to the time course, linear predictive coefficient (LPC) spectra, and spectrograms to verify that each stimulus has its own sound property (see Fig 1). Though the rat auditory system is not optimized for human vowel sound perception, we assumed that it is able to detect most of the sound stimuli since the frequency of sound belongs to the rat hearing range, that is, from 0.5 kHz to 64 kHz at 60 dB SPL [54].

### Data acquisition

**Fig 2:**
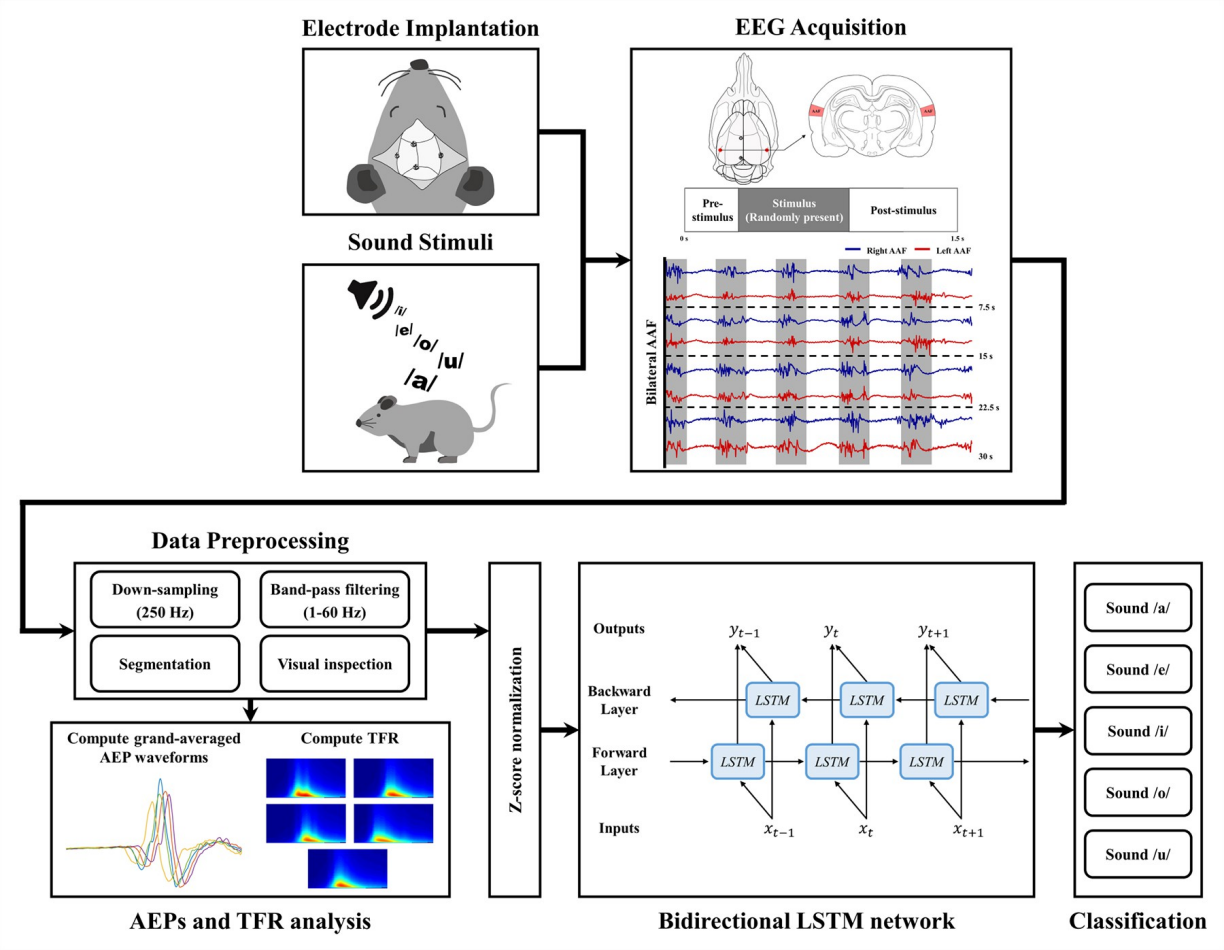
Schematic description of the experimental setup.

EEG signal responses to each vowel sound stimulus were acquired from the bilateral AAF after the one-week recovery period. First, the rats were anesthetized with isoflurane (5%) mixed with oxygen gas (0.6 L/min flow rate) for the induction. After the rats lost the righting reflex, the anesthesia was maintained with isoflurane (1.5%) via nosecone during recording to prevent contamination of EEG signals from motion artifacts. Next, a multi-pin connector was connected to a recording device (g.USBamp and g.HEADstage, g.tec medical engineering GmbH, Graz, Austria), which acquired signals at a 1200 Hz sampling frequency. The epidural EEG recording was performed for 1500 s per session, during which the five vowel speech sounds were randomly presented to each rat through the experimental speaker. Each speech stimulus appeared 130–150 times per stimulus in one session. To obtain sufficient EEG data, the recording session was repeated for five consecutive days. All recordings were performed in a soundproof booth to maximize SNR. A schematic diagram of the experiment is shown in Fig 2.

### EEG signal preprocessing and analysis

The acquired EEG signals were analyzed in response to each vowel sound using the FieldTrip toolbox [55] in MATLAB 2017b (Mathworks, Inc., MA, USA). In the first step, the raw EEG data were down sampled from 1200 to 250 Hz and band-pass filtered in the frequency range of 1 to 60 Hz. Then, the continuous EEG data were segmented into stimulus-specific trials with a 500 ms pre-stimulus period and 1500 ms post-stimulus period. Baseline correction was conducted based on the pre-stimulus period. To discard residual artifacts, contaminated trials were manually rejected using visual inspection methods.

After the pre-processing, the artefact-free EEG data were averaged for each speech stimulus to create the AEP waveforms and the TFR of the grand-averaged waveforms was calculated. TFR analysis was conducted based on Morlet wavelets to assess dynamic changes in spectral power over time for each speech stimulus. Utilizing grand-averaged AEP waveforms and their TFR, the time or frequency range that mainly reflected the brain response to speech stimulus was determined. In the case of TFR analysis, the analysis of variance (ANOVA) test and Bonferroni correction were performed to identify the statistical significance between the TFRs of EEG signals for each speech stimulus [48]. Through these results, the pre-processed EEG data was reorganized for later use for the purpose of classification. To ensure that the reconstructed data is meaningful, the time and frequency ranges of all EEG trials were restricted. The time range was set to 0.2–0.8 s and the frequency range was set to 1–60 Hz. After redefining the time and frequency ranges, all EEG trials were normalized using z-score normalization, a commonly used method to reduce variability among trials while maintaining a similar tendency within the trials [56, 57]. It is well known that the overall classification performance is improved following z-score normalization [56]. After this, the z-score normalized dataset was randomly shuffled and separated into a training set (90%) and test set (10%) to be used as inputs for deep learning and machine learning classifiers for speech recognition classification.

### Bidirectional long short-term memory networks

**Fig 3:**
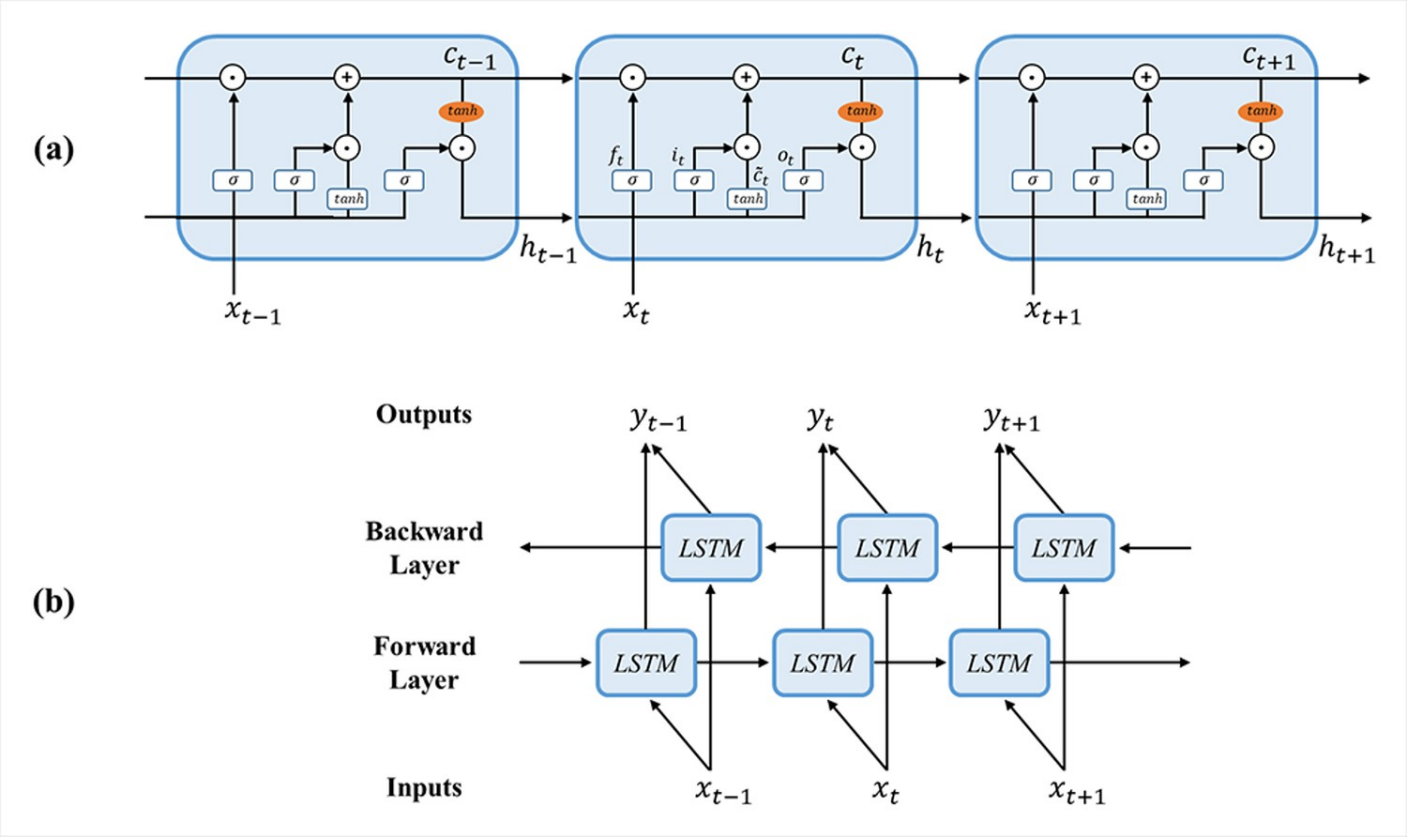
Structure of the BiLSTM network. (a) The structure of a long short-term memory (LSTM) cell and (b) architecture of bidirectional LSTM (BiLSTM) network.

LSTM is a special recurrent neural network (RNN) architecture that overcomes the vanishing/exploding gradient problem by incorporating gate structures that control the state of memory cells [46, 58]. For this reason, LSTM has shown stable and powerful performance for modeling long-term dependencies in a variety of temporal or sequential tasks [46, 58–61]. The structure of the LSTM is shown in Fig 3A. The main difference between conventional RNN and LSTM is the memory cell, *c*_*t*_, which can preserve the state information which is modulated by three kinds of self-parameterized gates: the input gate *i*_*t*_, forget gate *f*_*t*_, and output gate *o*_*t*_. The input gate *i*_*t*_ decides whether a new input will be accumulated in the memory cell; the forget gate *f*_*t*_ can discard the past status of the memory cell, *c*_*t*−1_; and the output gate *o*_*t*_ regulates the propagation of the output from the current memory cell *c*_*t*_ into the output response *h*_*t*_. The key processing of LSTM is described by the following equations:

$$i_{t}=\sigma (W_{i}x_{t}+R_{i}h_{t-1}+b_{i})$$ (1)

$$f_{t}=\sigma (W_{f}x_{t}+R_{f}h_{t-1}+b_{f})$$ (2)

$$o_{t}=\sigma (W_{o}x_{t}+R_{o}h_{t-1}+b_{o})$$ (3)

$${\tilde{c}}_{t}={tanh(W}_{c}x_{t}+R_{c}h_{t-1}+b_{c})$$ (4)

$$c_{t}=f_{t}⨀c_{t-1}+i_{t}⨀{\tilde{c}}_{t}$$ (5)

$$h_{t}=o_{t}⨀tanh(c_{t})$$ (6)

where σ and *tanh* are nonlinear activation functions. The logistic sigmoid function, defined as *σ*(x) = 1/(1+*e*^−*x*^) is utilized as the gate activation function, and the hyperbolic tangent function, tanh(*x*) = (*e*^*x*^−*e*^−*x*^)/(*e*^*x*^+*e*^−*x*^), is used as the block input and output activation function. Element-wise multiplication of two vectors is denoted by ⨀; *W*, *R* represent the weight matrices, and *b* denotes the bias vector, which are learnable parameters that control each gate.

LSTM is attested as a powerful structure for handling sequential data [59]; however, the standard LSTM captures only the past information from the sequence in the forward direction. BiLSTM was implemented to improve the structure. BiLSTM is a type of LSTM version of a bidirectional RNN [47, 62]. It has two layers of LSTM, as shown in Fig 3B; one processes information in the forward direction while another processes it in the backward direction. By accessing both past and future information, these structures can capture rich information from a sequence. Hence, the existing literatures shows that BiLSTM performs better than the standard LSTM in classifying EEG signals according to each task [63–67].

In this study, the BiLSTM network was used to classify five different vowel speech sounds using single-trial basis EEG signals. A BiLSTM layer containing 600 LSTM units was set and to avoid overfitting, the dropout ratio on the LSTM layers was set to 0.3 [68]. After the LSTM layers, the hidden states were concatenated into the fully connected layer with a softmax activation function, used for multiclass classification. Categorical cross entropy was adopted as the loss function with the ADAM optimizer [69] and the initial learning rate and learning rate decay were set to 1e-3 and 1e-6, respectively. Furthermore, the model was trained with 500 epochs and a batch size of 64. The learning curve reached a stable plateau within 500 epochs.

These hyper-parameters were adjusted to best fit the model to the data. A stratified 10-fold cross-validation (10-CV) was used to evaluate model performance. The k-fold cross-validation is an effective method to test the success rate of models used for classification and k = 10 is generally considered as the most reasonable parameter in applied machine learning [70].

The model was implemented using the Keras library [71] with TensorFlow backend [72] and the Scikit-Learn library [73] in Python.

### Machine learning classifiers

The performance of BiLSTM was compared with conventional machine learning classifiers: SVM with linear kernel (SVM_lin), SVM with radial basis function kernel (SVM_rbf), random forests (RF), NB, and KNN. SVM [74] aims to determine the optimally separated hyperplane by maximizing the margin, which is the distance between the support vectors. By using the kernel trick, SVM is capable of mapping feature space from low to high dimensions; therefore, it can efficiently perform linear classification and non-linear classification. RF [75] operates by constructing multiple decision trees during the training phase and generating the final class that combines the results of each decision tree. NB [76, 77] is a probabilistic classifier based on Bayes’ theorem and conditional probability which usually assume that all features are independent of each other. KNN [78] is a non-parametric approach that classifies the input based on the majority class of its k-nearest neighbors in the feature space. Usually, the k value is selected as an odd number to avoid tied classes. To train and evaluate the above machine learning models, the same 10-CV was used as in BiLSTM. All machine learning models were implemented using the Scikit-Learn library [73] in Python.

### Statistical analyses

All statistical analyses were performed using SPSS software (SPSS version 20.0, SPSS Inc., Armonk, NY, USA) and MATLAB software version 2017b (Mathworks, Inc., MA, USA). The data was analyzed with parametric statistics since all the data in the study showed a normal distribution in the Shapiro–Wilk test (*p* > 0.05). ANOVA was used to analyze the statistical significance of the TFRs according to the different vowel stimuli. In addition, a repeated-measures ANOVA was conducted to compare the performance of each classifier. Subsequently, pairwise comparisons using paired t-tests were performed between the BiLSTM network and other classical machine-learning classifiers and a Bonferroni correction was performed to adjust for the type I error rate inflation. The statistical significance of the *p*-value was set at 0.01, when comparing the TFR of EEG responses, while the significance level of the *p*-value was set at 0.05, when comparing the performance between the BiLSTM network and other machine learning classifiers.

## Results

### Auditory evoked potentials in response to vowel sounds

**Fig 4:**
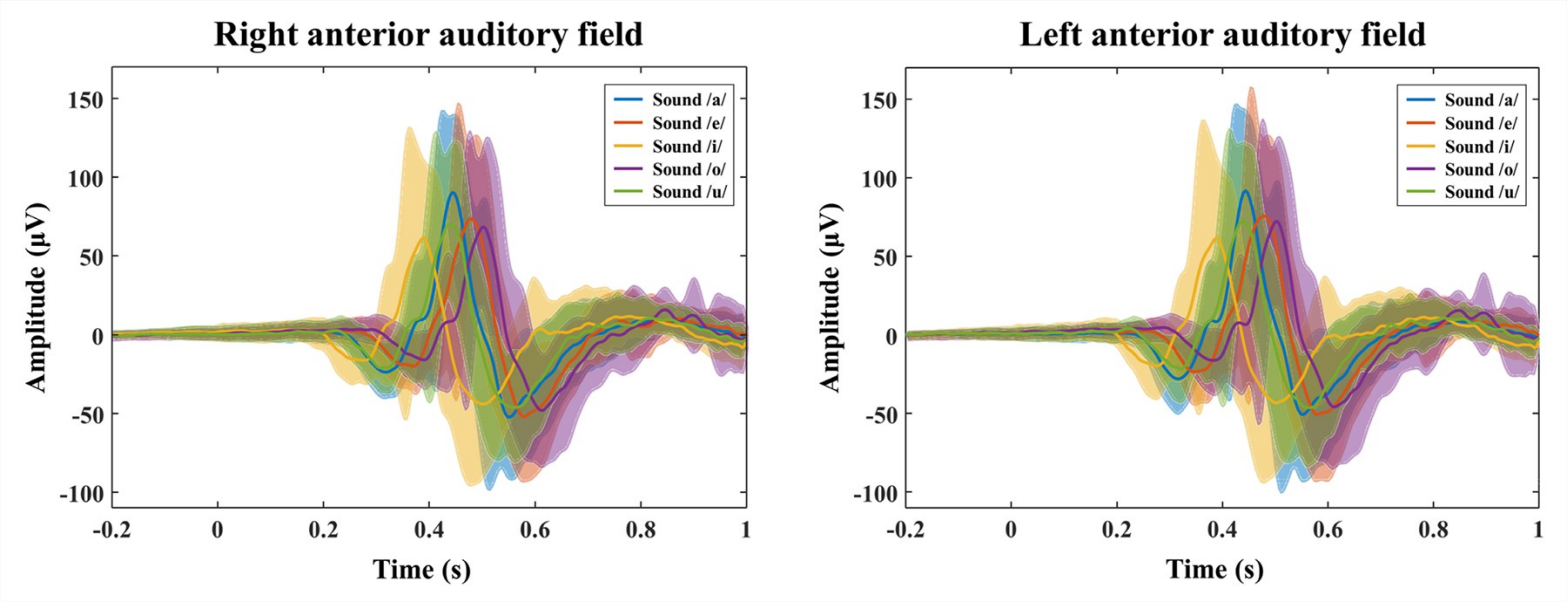
Averaged auditory evoked potentials (AEP) waveforms over the subjects to each vowel sound. AEPs were recorded on the right and left anterior auditory fields (AAFs). Overall, the neural responses were elicited 0.2–0.4 s after the sounds stimulus onset and showed different peak latencies and amplitudes depending on the vowel stimulus. The AEPs recorded on both AAFs were generally similar. The bold lines represent the averaged AEP waveforms, and the shaded areas represent the standard deviation.

A total of 19 Sprague-Dawley rats underwent epidural electrode implantation surgery, and all rats survived the surgical procedure. As a result, EEG responses to five English vowel sounds were recorded from 19 isoflurane-anesthetized rats. To extract the mean AEP waveforms, all the neural responses were averaged over the subjects for each stimulus. Fig 4 presents the averaged AEP waveforms for each vowel sound from bilateral AAF.

As expected, each categorical vowel sound evoked distinct neural activities in the bilateral AAF with varying peak amplitudes and latencies. The peak amplitude of AEPs, defined as the highest recorded voltage after the vowel stimuli, was smallest for /i/ (61.74 ㎶ in left AAF and 61.27 ㎶ in right AAF), while AEPs in response to /a/ showed the largest peak amplitudes (92.12 ㎶ in left AAF and 90.18 ㎶ in right AAF). The peak latency, defined as the duration from stimulus onset to the peak amplitude was approximately 0.39 s to 0.5 s, shortest in /i/ (0.39 s in left and right AAFs), and longest in the /o/ sound (0.51 s in left and right AAFs). As shown in Fig 4, similar AEP waveforms were observed from the left and right AAFs.

### Time-frequency analysis of the EEG signals

**Fig 5:**
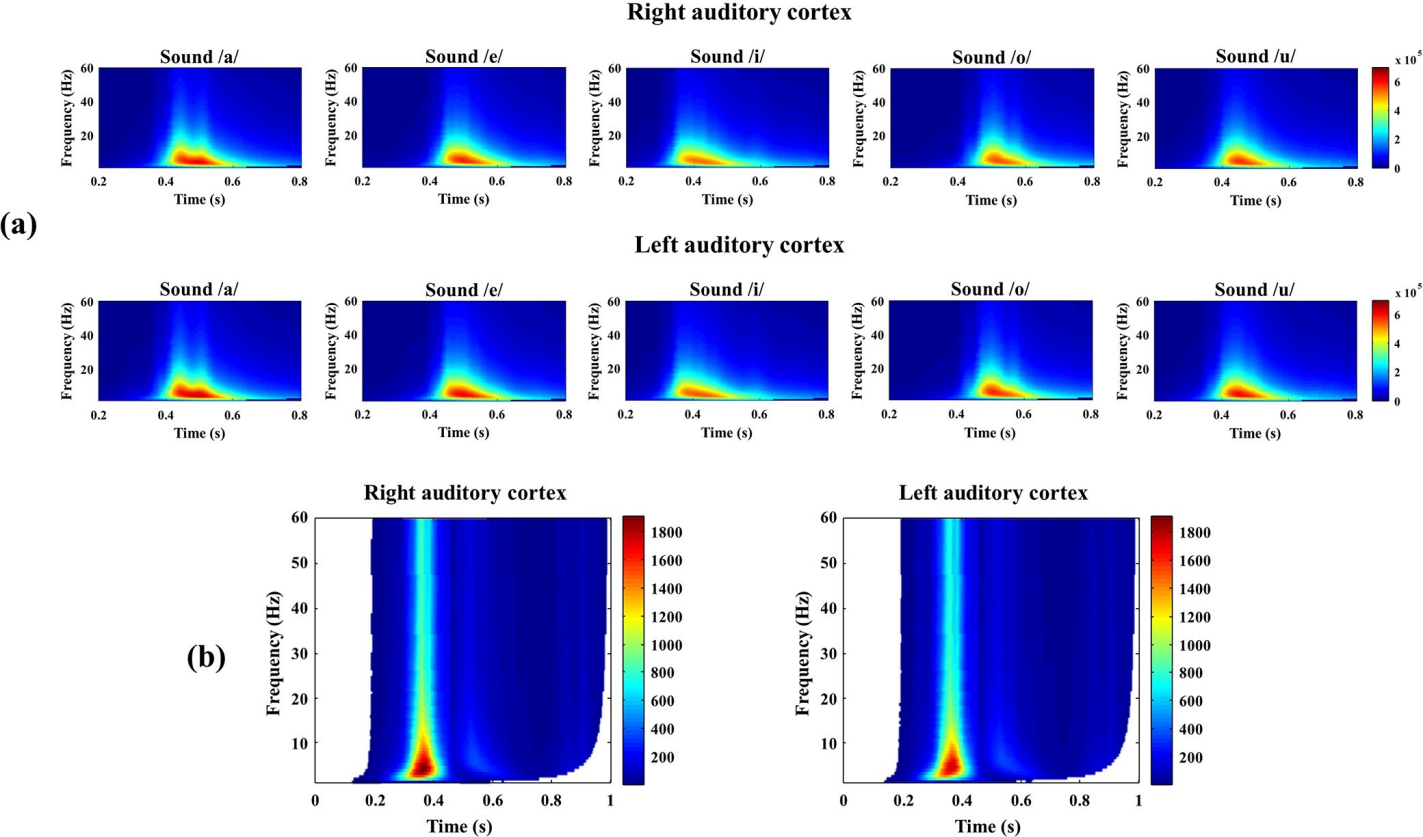
Subject-averaged time-frequency power results. (a) The time-frequency representation (TFR) of the event-related EEG signals (speech stimulation /a/, /e/, /i/, /o/, or /u/) over all subjects on the right and left anterior auditory fields. TFRs were plotted for the frequency range of 4 to 40 Hz, 0.2 to 0.8 s after each stimulus. One can observe high power activation in the low frequency band (especially in the delta, theta, and alpha band) between 0.3 and 0.6 s regardless of the sound stimulation. (b) Time-frequency regions with significant differences after the Bonferroni correction (*p* < 0.01 on the ANOVA test) are plotted. The color scale represents the F-values and non-significant regions are colored in white. Note that most of the brain responses between 0.2 and 0.8 s after the stimuli showed distinct neural responses across each vowel sound.

Time-frequency analysis is a powerful method for analyzing nonstationary EEG signals over a time-frequency plane and is used to provide qualitative information for the classification of EEG [79, 80]. Therefore, the TFR of the grand-averaged EEG was calculated for each sound to identify vowel recognition-related changes in the magnitude and phase of EEG oscillations at specific frequencies (Fig 5A). From the TFR analysis, high power activation was observed around the delta (1–4 Hz), theta (4–8 Hz), and alpha (8–12 Hz) band at 0.3–0.6 s from the stimulus onset, regardless of the speech sound stimulation.

In addition, an ANOVA test with a Bonferroni correction was conducted to analyze the statistically significant TFR components according to each vowel stimulus. Subsequently, the power of statistically significant areas (*p* < 0.01) was represented by the F-value (Fig 5B). In the analysis, most of the EEG frequency band from 0.2–0.8 s was significantly different according to the vowel stimuli. In addition, part of the TFR from 0.8–1 s was also statistically different for each stimulus. Considering the AEP waveforms and the results of the ANOVA tests, it was inferred that the AEPs from 0.2–0.8 s after the vowel stimulus were the most informative neural responses and were related with the vowel sound recognition.

### Model training and evaluation of the BiLSTM networks

**Fig 6:**
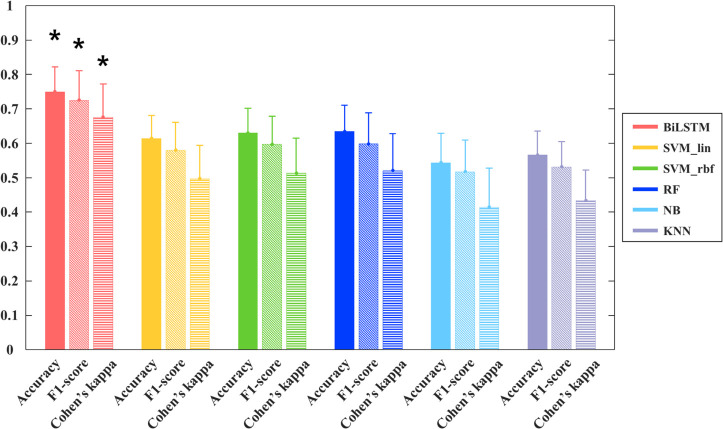
Comparison of the performance of the BiLSTM network and other conventional machine learning methods. The bar plots with standard deviation were drawn using the results of 10-fold cross-validation of each subject. Each bar represents accuracy (entire), f1-score (diagonal), and Cohen’s kappa statistic κ (horizon) of each classifier. Asterisk (*) above the bar plot indicates the significant differences (*p* < 0.01) between the performance of the BiLSTM and all other conventional machine learning methods. BiLSTM, bidirectional long short-term memory; SVM_lin, support vector machine with linear kernel; SVM_rbf, support vector machine with radial basis function kernel; RF, random forests; NB, naïve Bayes; KNN, k-nearest neighbors.

Based on the results of Fig 5B, EEG data that were band-pass filtered between 1–60 Hz with a time window of 0.2–0.8 s were selected. Then, the z-scores of the selected EEG data were used as the input to the BiLSTM network. All EEG data were divided into 10 folds within each subject to evaluate the BiLSTM networks. Therefore, the test performance was obtained per fold using the trained model with the remaining folds in a 10-CV scheme. The performance of the network was evaluated using metrics of accuracy, f1-score, and Cohen’s kappa statistic κ (Fig 6 and Table 1). The average five-class EEG discrimination accuracy of the BiLSTM network was 75.18 ± 7.06% and the f1-score was 0.74 ± 0.08. Cohen’s κ was 0.68 ± 0.09, which was interpreted as a moderate agreement [81].

**Table 1:** Overall performance of the BiLSTM network and other conventional machine learning methods.

Classifier	Accuracy (%)	F1-score	Cohen’s kappa (κ)
BiLSTM	75.18 ± 7.06	0.74 ± 0.08	0.68 ± 0.09
SVM_lin	61.47 ± 6.52	0.60 ± 0.08	0.50 ± 0.09
SVM_rbf	63.11 ± 7.04	0.62 ± 0.08	0.51 ± 0.1
RF	63.21 ± 7.41	0.62 ± 0.09	0.52 ± 0.1
NB	53.39 ± 8.40	0.52 ± 0.09	0.41 ± 0.11
KNN	56.80 ± 6.76	0.55 ± 0.07	0.43 ± 0.09

Data are presented as the mean ± standard deviation. BiLSTM, bidirectional long short-term memory; SVM_lin, support vector machine with linear kernel; SVM_rbf, support vector machine with radial basis function kernel; RF, random forests; NB, naïve Bayes; KNN, k-nearest neighbors.

**Fig 7:**
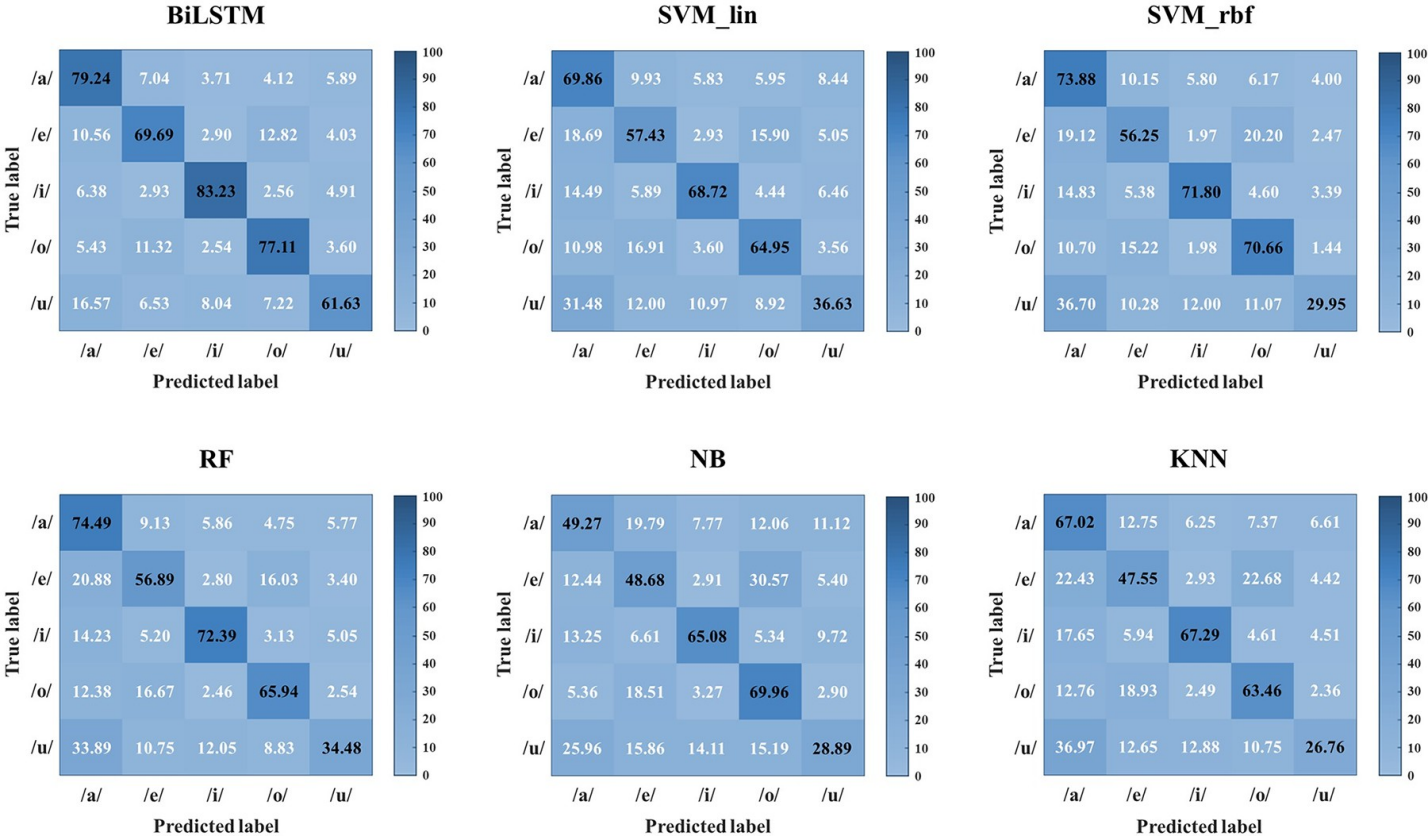
Confusion matrix for the BiLSTM network and other conventional machine learning classifiers. BiLSTM, bidirectional long short-term memory; SVM_lin, support vector machine with linear kernel; SVM_rbf, support vector machine with radial basis function kernel; RF, random forests; NB, naïve Bayes; KNN, k-nearest neighbors.

To analyze the performance of the BiLSTM network in more detail, the confusion matrix in Fig 7 was plotted. This indicated that many of the errors were due to the misclassification of the EEG responses to /u/ as /a/ and /e/ as /o/. However, the BiLSTM network classified most of the EEG responses with more than 50% accuracy, a high accuracy in the five-class EEG classification.

### Comparison of the BiLSTM network with other machine learning methods

To validate the effectiveness of the BiLSTM networks in classifying EEG for vowel sound recognition, the results were compared with those of other conventional machine learning methods. Fig 6 and Table 1 show the performance of the machine learning classifiers. The RF demonstrated the highest classification accuracy among the conventional machine learning algorithms (accuracy: 63.21 ± 7.41%, f1-score: 0.62 ± 0.09, and Cohen’s: 0.52 ± 0.1). In the statistical analysis, the classification performance of RF was not significantly higher than that of SVM_lin and SVM_rbf, while it showed higher performance when compared with those of NB and KNN. However, when the performance of conventional machine learning algorithms, including RF, was compared with BiLSTM, it was obvious that the BiLSTM network was superior for all the metrics used in the study (*p* < 0.01).

In the confusion matrix, conventional machine-learning algorithms cannot discriminate certain EEG responses well. In particular, all the conventional machine learning algorithms had difficulty distinguishing the sound /u/. It was noted that the algorithms showed a tendency to misclassify sound /u/ as /a/ on average 30% of the time (25.96% in NB to 36.97% in KNN), resulting in a decrease in the overall classification performance (Fig 7).

## Discussion

In this study, rat epidural EEG responses to five categorical vowel sounds (/a/, /e/, /i/, /o/, and /u/) were discriminated using the BiLSTM network. Five-class classifications of epidural EEG signals were performed on a single-trial basis, which is known to be challenging. To maximize learning performance, this study tried to determine specific EEG components that might be related to the recognition of speech sounds in the rat brain and utilized these EEG components as input features. As a result, a relatively high performance in classifying AEPs to five different vowel sounds was achieved using BiLSTM. A comparison of the classification performance of the BiLSTM network with other machine learning algorithms showed that the BiLSTM network outperformed other classical classifiers. These results indicate that the BiLSTM network trained with speech recognition-related EEG components reliably classifies AEPs to each categorical vowel sound with a high degree of accuracy. To our knowledge, LSTM networks have not been applied to the classification of EEG responses to auditory stimuli, and this is the first study to use a deep learning algorithm to analyze EEG signals from rat AAF.

Currently, only a few studies have used LSTM architecture to achieve state-of-the-art results in EEG-based classification. The LSTM architecture is suitable for EEG-based classification because its chain-like structure can capture the temporal sequence of EEG data [82]. In the beginning, research focused on improving the classification results through various LSTM architectures; however, the input features were still extracted manually, as in conventional machine learning methods [83, 84]. Tsiouris et al. evaluated the performance of diverse combinations of LSTM network elements in order to find the most efficient LSTM architectures for detecting epileptic seizures, thus obtaining near-perfect results in seizure prediction (100% sensitivity and 99.86% specificity) [83]. Because LSTM is a powerful structure for processing sequential data, there are also studies that use raw EEG data as input features with minimal preprocessing. As the LSTM network directly learns features from raw EEG data, the performance in emotion recognition studies improved by at least 12% [85], and the results of motor imagery classification studies also improved [86], when compared with other traditional feature extraction techniques. Moreover, the BiLSTM architecture was utilized for EEG-based classification because it can access information from both past and future states. Therefore, in detecting various brain states reflected in EEG data, such as seizure, sleep, etc. [63–67], the BiLSTM network generally outperformed the LSTM network that only captures past information from the sequence in the forward direction. For this reason, high performance has been reported in recent EEG-based classification using BiLSTM networks. Sharma et al. achieved 82.01% classification accuracy for four types of emotions based on the BiLSTM algorithm and higher-order statistics [87]. In addition, the BiLSTM networks successfully classified epilepsy types and sleep stages [88, 89].

Similar to previous studies, this study achieved comparatively good results using BiLSTM networks. The proposed algorithm successfully discriminated the EEG responses to five vowel sounds with high values of accuracy, f1-score, and Cohen’s κ of 75.18%, 74.43%, and 0.68, respectively. The value of Cohen’s κ for five-class classification is higher than that seen in most current studies [90]. As illustrated in Fig 6, the BiLSTM method produced the highest value for all metrics compared with the other machine learning methods. In addition, to determine the statistical difference in classification performance, repeated-measured ANOVA results were analyzed between BiLSTM and other classical machine learning methods using all the metric values. Through statistical analysis, it was determined that the classification performance of the BiLSTM network was significantly higher than that of other classical machine learning methods (*p* < 0.01). This result was also consistent with the confusion matrix. As shown in Fig 7, the BiLSTM network predicted the true labels of the five vowel sounds well, whereas classical machine learning methods did not. The prediction acquired through the conventional machine learning classifier was especially poor at classifying the /u/ sound; the /u/ sound was mainly misinterpreted as /a/. Even RF, which showed the best performance among the five conventional machine learning classifiers, had a classification rate of 34.48% for the /u/ sound, with a 33.89% misclassification rate of the /u/ sound as an /a/ sound. As can be seen in Fig 4, the /a/ and /u/ sounds had a similar peak latency, which is one of the main characteristics of AEP waveforms (peak latency of sound /a/: 0.448, peak latency of sound /u/: 0.444). When classification was performed based on minimally pre-processed single-trial EEG signals, it seems that such similarities could not be distinguished by conventional machine learning algorithms, whereas the BiLSTM network could distinguish them. Given that the BiLSTM network can simultaneously access all past and future contexts, rich information can be learned through this network. In addition, even though the features reflecting the characteristics of EEG responses to each vowel sound were extracted directly from the forward and backward directions of the LSTM layer, the classification performance was improved. In this study, we can derive good classification results using a simple BiLSTM architecture without an additional handcrafted feature extraction process.

Classifying the ERP responses to speech stimuli in a single trial is very challenging owing to the characteristics of the low SNR of EEG. Although one of the key advantages of the deep learning method is its ability to learn high-level features without hard-core feature extraction, we attempted to select the most relevant EEG signals related to speech recognition to achieve better performance. In this study, distinct AEP waveforms corresponding to each speech sound stimulus were observed with the high-power activation of the low-frequency band, including the delta, theta, and alpha bands, in the TFR analyses. Neural oscillations in the alpha band have been widely recognized to play an important role in auditory processing. Mazaheri et al. reported that the attenuation of alpha activity is closely related to the discrimination of auditory targets [91]. Staruß et al. proved that cortical alpha oscillations are a pivotal mechanism for selectively inhibiting the processing of noise to improve the auditory selective attention toward target signals [92]. Previously, we also found that alpha power was highly activated in bilateral temporal areas after specific sound stimuli that were statistically different in terms of the type of sound [48]. In addition, the delta and theta bands are known to be associated with shaping the segmentation and perceptual influence of acoustic information [93]. Although this study is based on animal experimental data, similar speech-related components, as compared to the previous studies on human subjects, were observed in the TFR analyses. Moreover, in the statistical analysis, all the EEG bands were found to be significant within 1 s after the stimuli and represented the EEG components related to sound perception. These results were somewhat different from those of previous studies, suggesting that only specific EEG bands, such as the alpha band, were related to sound perception. It is expected that even subtle changes across all the EEG band activities are recorded through the epidural EEG recording, because it provides a higher SNR by reducing volume conduction and eliminating the artifacts that are inherent to extracranial EEG recordings.

In this study, the speech sound recognition related EEG components in rats were determined and the AEP components were successfully classified using the BiLSTM network. However, this study had some limitations. First, the number of subjects included was too small, especially for deep learning. Moreover, this study did not evaluate each classifier’s performance with external validation, but instead used 10-CV to overcome the limited sample sizes. Besides, we cannot rule out the possibility that the rat’s auditory system responds continuously to sound, since only a single utterance of each vowel sound was used in this study. In addition, the acquired EEG responses were affected by the anesthetic effects. Although minimal anesthetic dose was used, frequency slowing with increase in delta power is a typical finding of EEG changes after isoflurane inhalation [94]. Therefore, the vowel recognition EEG components suggested in this study may be different from EEG signals acquired from rats that are awake. However, we believe that the quality of the EEG signal is good enough since we recorded EEG through epidural electrode implantation, and it was not contaminated by motion artifacts.

## Conclusions

In conclusion, this study extracted meaningful neural components related to categorical speech perception. Furthermore, based on the characteristics of the LSTM networks, it was proved that the BiLSTM network was suitable for classifying EEG responses with minimally pre-processed AEPs. Since this study is pioneer research with animal data, it may not be directly transferable to other practical applications such as brain-computer interfaces or alternative communication aids for humans. Therefore, future studies with human EEG data are required to verify the effectiveness of the BiLSTM network in classifying auditory EEG-based speech recognition. Additionally, it needs to be re-evaluated for optimal parameter tuning and feature extraction. It is expected that this study will provide a novel approach for analyzing EEG signals and as well as valuable information regarding the mechanisms of speech perception and recognition in the brain.

## Supporting information

S1 Dataset (TXT)

S1 File (ZIP)
